# Quaking-induced conversion of prion protein on a thermal mixer accelerates detection in brains infected with transmissible spongiform encephalopathy agents

**DOI:** 10.1371/journal.pone.0225904

**Published:** 2019-12-12

**Authors:** Nadine Kaelber, Cyrus Bett, David M. Asher, Luisa Gregori

**Affiliations:** Food and Drug Administration, Center for Biologics Evaluation and Research, Silver Spring, Maryland, United States of America; Van Andel Institute, UNITED STATES

## Abstract

Detection of misfolded prion protein, PrP^TSE^, in biological samples is important to develop antemortem tests for transmissible spongiform encephalopathies (TSEs). The real-time quaking-induced conversion (RT-QuIC) assay detects PrP^TSE^ but requires dedicated equipment and relatively long incubation times when applied to samples containing extremely low levels of PrP^TSE^. It was shown that a microplate shaker with heated top (Thermomixer-C) accelerated amplification of PrP^TSE^ in brain suspensions of 263K scrapie and sporadic Creutzfeldt-Jakob disease (sCJD). We expanded the investigation to include TSE agents previously untested, including chronic wasting disease (CWD), macaque-adapted variant CJD (vCJD) and human vCJD, and we further characterized the assays conducted at 42°C and 55°C. PrP^TSE^ from all brains containing the TSE agents were successfully amplified using a truncated hamster recombinant protein except for human vCJD which required truncated bank vole recombinant protein. We compared assays conducted at 42°C on Thermomixer-C, Thermomixer-R (without heated top) and on a fluorimeter used for RT-QuIC. QuIC on Thermomixer-R achieved in only 18 hours assay sensitivity similar to that of RT-QuIC read at 60 hours (or 48 hours with sCJD). QuIC on Thermomixer-C required 24 hours to complete and the endpoint titers of some TSEs were 10-fold lower than those obtained with RT-QuIC and Thermomixer-R. Conversely, at 55°C, the reactions with sCJD and CWD on Thermomixer-C achieved the same sensitivity as with RT-QuIC but in shorter times. Human vCJD samples tested at higher temperatures gave rise to high reactivity in wells containing normal control samples. Similarly, reactions on Thermomixer-R were unsuitable at 55°C. The main disadvantage of Thermomixers is that they cannot track formation of PrP fibrils in real time, a feature useful in some applications. The main advantages of Thermomixers are that they need shorter reaction times to detect PrP^TSE^, are easier to use, involve more robust equipment, and are relatively affordable. Improvements to QuIC using thermal mixers may help develop accessible antemortem TSE tests.

## Introduction

### Transmissible spongiform encephalopathies are transmitted iatrogenically

Transmissible spongiform encephalopathies (TSEs) or prion diseases are rare fatal infections characterized by the accumulation of abnormally folded prion protein, PrP^TSE^, in affected organs and tissues [1, 2]. Expression of the normal “cellular” prion protein, PrP^C^, while not necessary to sustain life, must play an essential role in TSEs in that mice and cattle genetically modified to lack PrP^C^ cannot be infected with a TSE agent [3, 4]. The normal cellular functions of PrP^C^ remain elusive. During the progression of the diseases, normal α-helix-rich PrP^C^ is converted to PrP^TSE^, a β-sheet-enriched relatively insoluble isoform that resists digestion with proteolytic enzymes. The cellular events that trigger this conversion as well as the mechanism by which PrP^TSE^ propagates in vivo are still not fully understood.

Iatrogenic transmissions of TSE infections from blood and tissues of asymptomatic donors are rare but have occurred and, thus, iatrogenic TSEs pose a demonstrated risk to public health [5–7]. Detection of infected asymptomatic TSE carriers is important to prevent iatrogenic TSE and to enhance TSE surveillance and epidemiology. The target analyte for all candidate antemortem TSE assays currently under development is PrP^TSE^, the only known protein biomarker of TSE infectivity [8].

### *In vitro* methods to detect misfolded prion protein

Protein misfolding cyclic amplification (PMCA [9]) and real-time quaking-induced conversion (RT-QuIC [10]) are two highly sensitive and specific in vitro methods to detect low concentrations of PrP^TSE^. Both methods rely on the ability of PrP^TSE^ to catalyze in vitro the conversion of the normal prion protein to a misfolded amyloid form of the protein [9, 10]. In RT-QuIC, PrP^TSE^ (“seed”) in a sample catalyzes the conversion of purified recombinant prion protein monomers (“substrate”) into fibrils using cycles of vigorous shaking to break up fibrils and create multiple new seeding units [10]. This key event is visualized by the selective binding of fibrils, but not soluble monomeric recombinant PrP, to the fluorescent dye Thioflavin T. Formation of new fibrils is detected in real time as an increase in fluorescence [11]. The output of RT-QuIC can be measured semi-quantitatively as doses of PrP^TSE^ expressed as 50-percent seeding activity (SD_50_) in a manner analogous to other quantal calculations of 50-percent endpoint titers derived from ratios of infected versus uninfected animals or cell cultures exposed to serial dilutions of conventional viruses [12].

### QuIC on a thermal mixer

One practical challenge of the current RT-QuIC assay is its relatively long lag time when tested at 42°C, presumably required to initiate assembly of fibrils and to propagate them to detectable amounts. In some cases, assaying biological materials containing minute amounts of PrP^TSE^ by RT-QuIC can take 40–50 hours or longer [13–15]. Recent modifications of the method conducting the reaction at 55°C and using truncated prion protein as the substrate, have markedly reduced lag times [16–19]. However, RT-QuIC assay time is a function of PrP^TSE^ concentration in the test materials, requiring longer times to detect low levels of the abnormal protein. Thus, while the recent improvements made RT-QuIC more suitable for diagnostic applications, its use to screen samples from blood and tissue donors will likely require shorter turnaround times. Thus, further reduction of reaction times is desirable. Caughey’s group first reported using Thermomixer-R for QuIC with 263K scrapie hamster brain seed amplified in tubes and detected by Western blot [20]. The same group later used a heated plate shaker analogous to the Thermomixer and showed shortened reaction times with 263K scrapie but also increased fluorescence signals in negative control wells, consistent with our results [16]. Other investigators have used Thermomixer-C at 42°C with cerebral spinal fluid samples from sCJD patients and reported faster reaction times without reduced sensitivity [21]. We investigated whether such acceleration was a general property of thermal mixers and expanded the study to include TSE agents not previously examined. We also report for the first time the effect of increasing the temperature of QuIC reactions to 55°C on thermal mixers. The final goal of this study was to establish assay conditions that reduced the amplification times needed to detect low levels of PrP^TSE^ in infected samples while maintaining the high sensitivity and specificity that needed for future reliable antemortem tests using blood or other readily accessible biological fluids.

## Materials and methods

### Ethic statement

All brain homogenate samples used in this study were repository samples collected post-mortem. Human brain homogenate samples were prepared by the UK National Institute for Biological Standards and Controls. These sample are anonymized and are WHO reference reagents. We purchased the samples. Macaque brain homogenate was produced at FDA under FDA IACUC approval #2009–14. Deer brain homogenate was from an FDA IACUC-approved protocol #2013–21. Hamster brain homogenate was from an FDA IACUC-approved protocol #2014–14.

### Brain homogenates

We obtained human vCJD (Ru98/148), sCJD (Ru99/009) and normal human (Ru97/03) brain homogenates from the World Health Organization (WHO) Human TSE Biological Reference Materials collection at the CJD Resource Centre, National Institute for Biological Standards and Control, Potters Bar, UK [22, 23]. These materials were provided as 10% w/v homogenates in 5% sucrose. We obtained brain homogenates (10% w/v in PBS) from whitetail deer with CWD and uninfected deer as a generous gift from the Prion Research Laboratory, National Wildlife Health Center, United States Geological Survey (USGS), Madison, WI.

We prepared brain homogenates from hamsters infected with the 263K strain of scrapie agent and uninfected hamsters. We also prepared a pool of homogenized brains from three vCJD-infected cynomolgus macaques and a control preparation of uninfected macaque brain [24, 25]. We homogenized all brains as 10% w/v suspensions in PBS pH 7.4 using glass beads in a bead-beater (BioSpec Products, Bartlesville, OK) by vigorously shaking twice for 1.5 min each and holding tubes on ice for 5 min between homogenizations.

### RT-QuIC assay

We performed RT-QuIC assays according to a protocol previously described [26] except when otherwise stated. Gain in the fluorimeter was set at around to 2000 for all tests. We purified two truncated prion protein peptides: Syrian golden hamster (Ha-recPrP [residues 90–231]) and bank vole (BV-recPrP [residues 90–230]) [11, 12] to serve as substrates. *E*. *coli* cells expressing the two proteins were a generous gift of Byron Caughey (Rocky Mountain Laboratory, National Institute of Allergy and Infectious Diseases, National Institutes of Health, Hamilton, MT). Briefly, we grew bacteria with pET41 vector containing the DNA sequence for each recombinant prion protein in Luria broth medium with kanamycin and chloramphenicol. We induced protein expression with the Overnight Express Autoinduction system 1 (EMD Millipore, Billerica, MA) and prepared inclusion bodies by cell lysis with Bug Buster Master Mix or Bug Buster Plus Lysonase Kit (EMD Millipore, Billerica, MA). We purified recombinant prion proteins (recPrP) from inclusion bodies on a Ni-nitrilotriacetic acid Superflow resin chromatographic column (Qiagen, Hilden, Germany) by gradient refolding in guanidine HCl followed by gradient elution in imidazole buffer using an AKTA Fast Protein Liquid Chromatography system (GE Healthcare Bio-Sciences, Pittsburgh, PA). We dialyzed the eluted recPrP in 10-mM sodium phosphate buffer (pH 5.8) and filtered it through a 0.22-μm syringe filter (Millipore). We determined protein concentrations by measuring absorbance at 280 nm, using 1.44 mg/ml as the extinction coefficient for the proteins and adjusted concentrations to approximately 0.6 mg/ml before storing aliquots of protein at -80°C.

We prepared serial dilutions of each infected sample in dilution buffer (0.1% SDS with 1x N2 supplement in PBS) and initiated the seeded reaction with 2 μl of sample mixed with 98 μl of cocktail in each well of a 96-well black plate. The cocktail contained recombinant PrP (Ha-recPrP or BV-recPrP) in 10-mM phosphate buffer (pH 7.4) with 300-mM NaCl, 1-mM ethylenediaminetetraacetic acid tetrasodium salt (EDTA) and 10-μM Thioflavin T dye. Typically, 0.1 mg/ml of substrate is used in RT-QuIC. We found that using 0.05 mg/ml recombinant proteins did not affect reaction times or assay sensitivities. However, detection of human vCJD brain homogenate on Thermomixer-C required BV-recPrP at 0.1 mg/ml. We tested each sample in four wells and repeated each test at least three independent times. We then incubated each plate in a FLUOstar Omega plate reader (BMG Labtech, Cary, NC) at 42°C. We also tested selected brain samples at 55°C, shaking at 700 rpm in cycles of one min on and one min off.

### QuIC assay on Thermomixers

Two commercial thermal mixers, Thermomixers-R and C (Eppendorf, Hamburg, Germany), were fitted with adaptors to accommodate 96-well microplates. The two mixers differed in that Thermomixer-C was equipped with a heated top to reduce condensation of liquid in the wells; the Thermomixer-R has an unheated top. We prepared identical microplates containing serial dilutions of infected brain homogenates as described above. We assayed each sample in four replicate wells in at least three independent experiments. We set Thermomixers to shake at 700 rpm for one min on and one min off at 42°C or 55°C. We removed the microplates from the shakers at the indicated times and read the signals in a FLUOstar Omega fluorimeter as indicated above.

### Data analysis

We calculated the average fluorescent signals from four-well replicates for each independent experiment, reported standard error of the mean (SEM) values, and plotted relative fluorescence units (rfu) versus log_10_ dilutions of the brain homogenates using GraphPad Prism software (GraphPad, La Jolla, CA).

Seeding activity or seeding dose relative to 2 μl of sample was calculated independently from each experiment using the Spearman and Karber method [12, 27]; the 50% seeding dose (SD_50_) was the calculated 50% endpoint dilution at which at least one lower dilution had 100% positive replicate wells and at least one higher dilution had no positive replicates. We considered a well to be positive if the fluorescence at the end of the reaction exceeded the cut-off value for that instrument (see below). We averaged SD_50_ for each experiment and reported that value with the standard deviation in Tables 1 and 2. We used a t-test (p<0.05) to establish statistical differences among SD_50_ values.

**Table 1:** Comparison of the 50-percent seeding doses (SD_50_) at 42°C.

	RT-QuIC 18 hr	RT-QuIC 60 hr	QuIC-R 18 hr	QuIC-C 24 hr
263K scrapie hamster	6.5 ± 0.5	9.7 ± 0.6	10.1 ± 0.4	8.7 ± 0.6
CWD deer	4.6 ± 0.8	9.1 ± 0.5	9.0 ± 0.7	8.1 ± 0.5
Macaque vCJD	6.7 ± 0.9	7.8 ± 0.4	8.4 ± 0.8	8.3 ± 0.1
Human sCJD	7.7 ± 0.3	9.7 ± 0.6 (48 hr)	9.7 ± 1.0	8.7 ± 0.1
Human vCJD	-	7.1 ± 0.7	6.7 ± 0.2	6.6 ± 0.3

Comparison of SD_50_ determined for each TSE brain homogenate tested using various assay platforms at 42°C: RT-QuIC at 18 hours and 60 hours, QuIC-R at 18 hours and QuIC-C at 24 hours. SD_50_ were calculated independently for each experiment; the averages and standard deviations were calculated from at least three independent experiments per condition.

**Table 2:** Comparison of SD_50_ at 55°C.

	RT-QuIC 8 hr	RT-QuIC 24 hr	RT-QuIC 36 hr	QuIC-C 8 hr	QuIC-C 24 hr
Human sCJD	9.3 ± 0.8	12.2 ± 0.6	-	12.0 ± 0.2	-
CWD deer	6.1 ± 0.4	8.1 ± 0.2	8.5 ± 0.2	7.6 ± 0.4	8.8 ± 0.3

Comparison of SD_50_ determined for CWD and sCJD brain homogenates tested using two assay platforms. The reactions were performed at 55°C. SD_50_ were calculated independently for each experiment; the averages and standard deviations were calculated from at least three independent experiments per condition.

### Reaction background and cut-off values

To determine the background for each instrument, we assayed three replicates (in four wells each) of normal human, hamster, and macaque brain homogenates, averaged the fluorescent signals from all these negative control wells, and calculated the standard deviation. The reaction cut-off values were calculated as the background plus three-times the standard deviation per each instrument. The background for RT-QuIC reaction at 60 hours was 43,000 rfu and the reaction cut-off was 64,000 rfu. The average background value for QuIC-R was 55,000 rfu and the reaction cut-off was 85,000 rfu. The average background value for QuIC-C was 21,000 rfu and the reaction cut-off was 33,000 rfu. We used these cut-off values to establish reactive/nonreactive wells for SD_50_ calculations.

## Results

### QuIC amplification using Thermomixer

**Fig 1:**
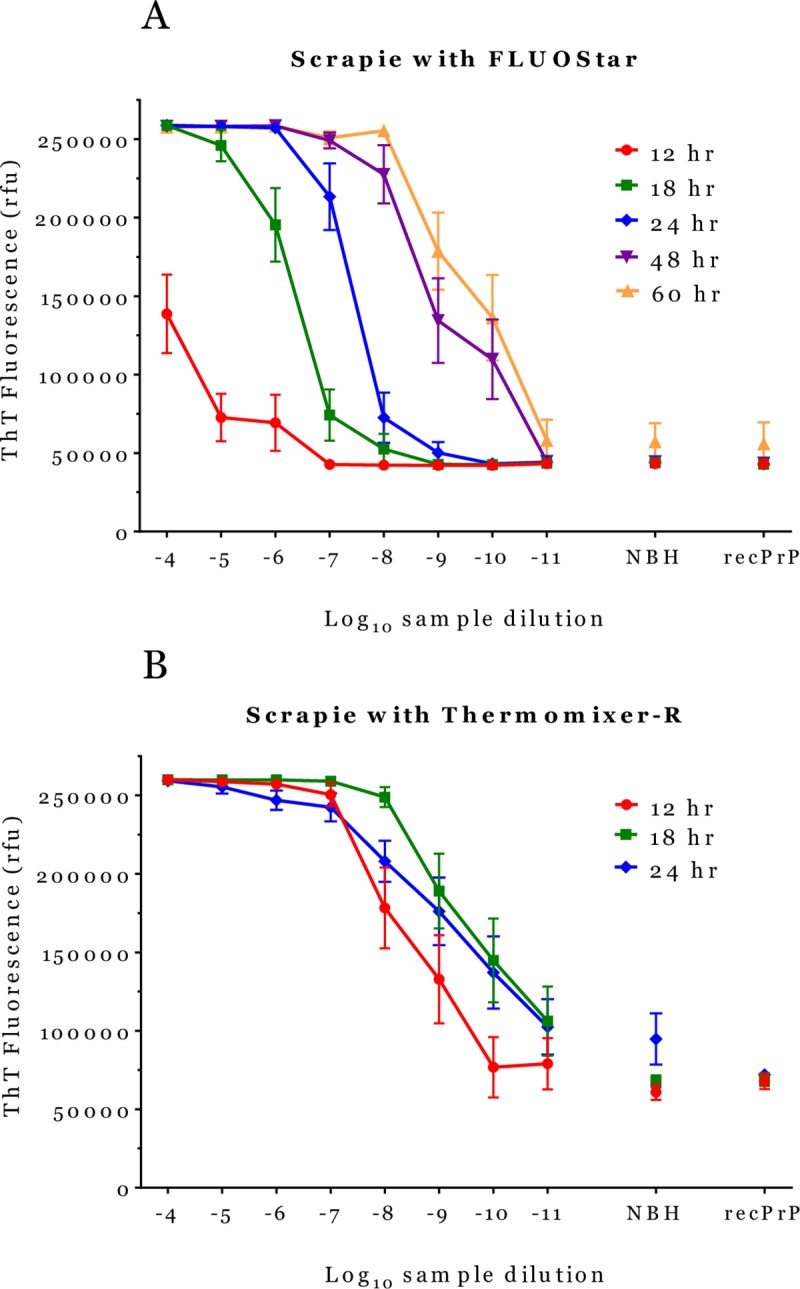
Amplification reactions with scrapie-infected hamster brain homogenates. Results in Fig 1 are average fluorescent signals ± standard errors of the mean (SEM) for reactions at 42°C. The maximum fluorescence intensity was 260,000 relative fluorescent units (rfu) for all experiments. Fig 1A. RT-QuIC was conducted with scrapie-infected hamster brain homogenate serially diluted from 10^−4^ to 10^−11^ (10% w/v = 10^−1^). Fluorescence values for each dilution (4 wells/dilution) were measured with a FLUOstar Omega fluorimeter. Fluorescence readings were reported at different times as indicated. We reported fluorescent signals for control samples at the indicated time points. Fig 1B. QuIC on a Thermomixer-R conducted as in Fig 1A. The same microplate was read on an external fluorimeter at the indicated times and the average signal at each dilution was reported with its SEM. NBH = Ha-recPrP substrate seeded with normal hamster brain diluted 10^−4^. recPrP = Hamster recombinant PrP unseeded.

We conducted QuIC using a Thermomixer-R, the cold-top shaker, (abbreviated QuIC-R) and a FLUOstar Omega fluorimeter (the device used to develop the original RT-QuIC) at 42°C. We first compared the two platforms by parallel tests using 263K scrapie hamster brain homogenate to seed truncated hamster recombinant prion protein (Ha-recPrP) substrate. Fig 1 shows results with FLUOstar Omega (Fig 1A) and Thermomixer-R (Fig 1B). At each indicated time, averaged fluorescent signals were plotted versus the log_10_ dilution of brain homogenates. After 12 hours of original RT-QuIC, only a few wells containing the highest concentrations of scrapie brain homogenate had become reactive. The RT-QuIC signals increased with time until 48 and 60 hours when the fluorescence reached plateau and the reactions were considered complete. QuIC on Thermomixer-R reactions were completed by 18 hours; prolonging the QuIC-R assay reaction times to 24 hours did not increase sensitivity (Fig 1B). As controls for specificity, we tested Ha-recPrP seeded with normal hamster brain homogenate (NBH) diluted 10^−4^ and the same cocktail and recombinant PrP but without adding brain homogenate.

### Accelerated amplification of PrP^TSE^ in 263K scrapie

**Fig 2:**
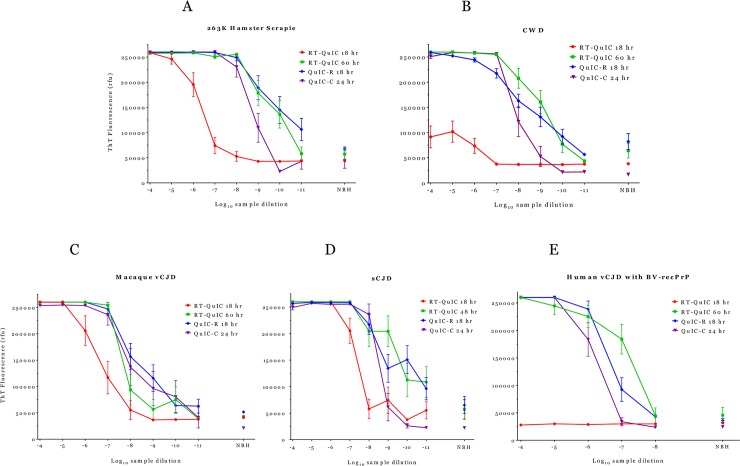
Amplification reactions at 42°C. Comparison of amplifications at 42°C with five TSE-infected brain suspensions as seeds. We used Ha-recPrP substrate to amplify PrP^TSE^ from scrapie (Fig 2A), CWD (Fig 2B), macaque-adapted vCJD (Fig 2C) and sCJD (Fig 2D) brain suspensions. BV-recPrP substrate amplified human vCJD brain seed (Fig 2E). RT-QuIC fluorescence signals were recorded only at 18 hours and 60 hours (48 hours for sCJD). QuIC-R and QuIC-C signals were recorded at 18 hours and 24 hours, respectively. NBH = recombinant PrP (hamster or bank vole) seeded with normal brain diluted 10^−4^.

Next, we tested brains infected with other TSE agents. We also evaluated QuIC conducted on Thermomixer-C (abbreviated QuIC-C). Fig 2A directly compares results from serial dilutions of scrapie brain homogenate using QuIC-R terminated at 18 hours, QuIC-C terminated at 24 hours, and RT-QuIC values recorded at both 18 and 60 hours. We calculated SD_50_, as a measure of assay sensitivity, on the three platforms (Table 1) and confirmed that QuIC-R had the same sensitivity as RT-QuIC. QuIC-C at 24 hours had SD_50_ 10-fold lower than those on the other two platforms (Table 1). However, SD_50_ values for QuIC-C and RT-QuIC were not statistically different (P<0.05) while SD_50_ for QuIC-C differed from that for QuIC-R (p = 0.018).

### Accelerated amplification of PrP^TSE^ in chronic wasting disease

Only high concentrations of CWD-infected deer brain homogenates showed fluorescent signals with RT-QuIC at 18 hours and reactions became complete at 60 hours (Fig 2B) consistent with previously reported reaction times [28]. In contrast, QuIC-R achieved maximum sensitivity at 18 hours and QuIC-C reactions reached endpoints by 24 hours not improving at longer times. The average background signals with normal control brain homogenates with QuIC-R and RT-QuIC were higher than the cut-off values. This high average fluorescence signals resulted from two out of 16 control wells that were reactive on both assay platforms. Not all normal deer brain homogenates we tested showed false positive reactions. It is possible that differences in sample storage conditions and tissue processing or other unknown factors caused the observed effect. SD_50_ of QuIC-C with CWD deer brain was 10-fold lower than those on the other two platforms (Table 1) and statistically different from that of RT-QuIC (p = 0.02) but not QuIC-R (p = 0.058).

### Accelerated amplification of PrP^TSE^ in macaque-adapted variant Creutzfeldt-Jakob disease

Ha-recPrP substrate seeded with macaque-adapted vCJD brain showed overlapping curves with QuIC-R and QuIC-C at endpoint. The average fluorescent signals on the two thermomixers tested with 10^−8^ and 10^−9^ dilutions of brain homogenate were higher than the signals with RT-QuIC for the same dilutions, suggesting an overall improvement in sensitivity with QuIC reactions for this agent (Fig 2C). Different from other agents tested, RT-QuIC conversion of Ha-recPrP into fibrils by macaque-adapted vCJD was relatively fast and the curve at 18 hours was close to the final curve at 60 hours. Although QuIC reactions performed better than RT-QuIC at higher brain homogenate dilutions, SD_50_ values for all three platforms were statistically equivalent (Table 1).

### Accelerated amplification of PrP^TSE^ in human sporadic Creutzfeldt-Jakob disease

Human sCJD brain homogenate seeded Ha-recPrP substrate at 42°C (Fig 2D). Results with RT-QuIC at 48 hours and QuIC-R at 18 hours were similar, while QuIC-C generated lower signals with high sCJD brain dilutions. We reported the RT-QuIC with human sCJD brain at 48 hours when the reaction had reached endpoint and fluorescence did not increase after that. Similar to macaque-adapted vCJD, human sCJD on RT-QuIC also seeded Ha-recPrP rapidly, generating fluorescent signals at 18 hours close to those at 60 hours. Nevertheless, even those seeds aggregating the substrate rapidly showed measurable and reproducible accelerated reactions on both Thermomixers. The SD_50_ on QuIC-C was 10-times lower than SD_50_ on the other two assay platforms (Table 1) but the three SD_50_ values were statistically equivalent.

### Accelerated amplification of PrP^TSE^ in human variant Creutzfeldt-Jakob disease

Human vCJD brain seeded truncated BV-recPrP efficiently but did not convert either truncated or full-length Ha-recPrP substrates as previously reported by others [29]. RT-QuIC generated no detectable PrP^TSE^ signal in 18 hours but reached completion at 60 hours (Fig 2E). QuIC-R accelerated PrP^TSE^ fibril formation and the reaction reached plateau by 18 hours, generating fluorescent signals close to those achieved with RT-QuIC. The average SD_50_ values for the three assays were similar and statistically equivalent (Table 1).

### Amplification reactions of PrP^TSE^ at 55°C

**Fig 3:**
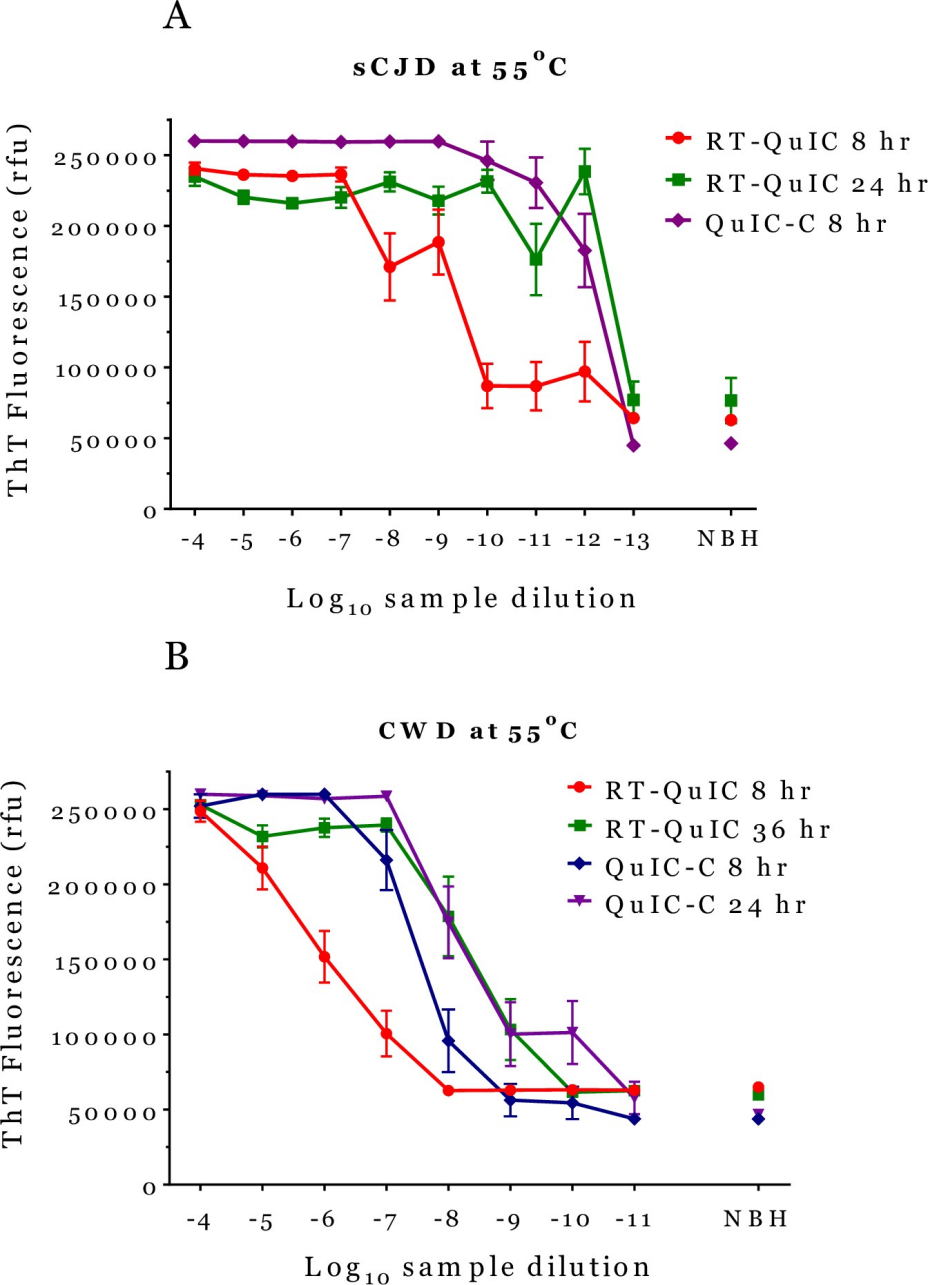
Amplification reactions at 55°C. Comparison of amplifications at 55°C with CWD and sCJD-infected brain suspensions as seeds at various reaction times. Fig 3A represents the average fluorescent signals and SEM with sCJD brain homogenates amplified with QuIC-C on Thermomixer-C at 8 hours and RT-QuIC on FLUOstar Omega at 8 hours and 24 hours. Fig 3B represents the average fluorescent signals and SEM with CWD brain homogenates amplified with QuIC-C on Thermomixer-C at 8 hours and 24 hours and RT-QuIC on FLUOstar Omega at 8 hours and 36 hours. NBH = hamster recombinant PrP seeded with normal brain diluted 10^−4^.

We investigated the performance of the three assay platforms at 55°C because the amplification reaction accelerates at higher temperatures [16–19]. We compared brain homogenates infected with CWD, sCJD and vCJD, three naturally occurring infections, tested with QuIC-C and RT-QuIC using truncated recombinant substrates. Thermomixer-R results had to be excluded because of excess condensation in the wells causing unreliable values. As expected, the reaction times with CWD and sCJD samples were shortened on both assay platforms. Fig 3A shows RT-QuIC results with sCJD at 8 hours and 24 hours when the reaction reached plateau. QuIC-C reached the same endpoint at only 8 hours with no improvement after longer reaction times. Statistical analysis confirmed no difference in the SD_50_ values at the endpoint for both assay platforms (Table 2).

Next, we seeded Ha-recPrP with CWD brain homogenate assayed with RT-QuIC, and analyzed the results at 8 hours, 24 hours (not shown in the Fig 3B) and at 36-hour endpoint (Fig 3B). Table 2 shows SD_50_ of RT-QuIC at 24 hours increased from 8.1 ± 0.2 at 24 hours to 8.5 ± 0.2 at 36 hours, which was the reaction endpoint. QuIC-C at 8 hours had a higher sensitivity than RT-QuIC at 8 hours (SD_50_ 7.6 versus 6.1) and reached the endpoint at 24 hours. Statistical analysis of SD_50_ confirmed that RT-QuIC and QuIC-C were different at 8 hours (p = 0.005) as were RT-QuIC and QuIC-C at 24 hours (P = 0.0042) but the two SD_50_ were statistically equivalent at the endpoint. Thus, although not as dramatic as results at 42°C, the trend confirmed that QuIC-C is faster than RT-QuIC when reactions are conducted at 55°C.

Tests with vCJD samples using truncated BV-recPrP at 55°C and 50°C failed because truncated BV-recPrP was unstable at those temperatures and gave rise to high non-specific fluorescence within a few hours of reaction.

## Discussion

### Thermomixer accelerated the rates of amplification with all TSE agents tested

We demonstrated accelerated rates of fibril formation for all TSE-infected brains tested at 42°C. QuIC on Thermomixer-R (unheated-top shaker) reached endpoint in 18 hours, and Thermomixer-C (heated-top shaker) in 24 hours compared to RT-QuIC which required 60 hours (48 hours with sCJD). When tested CWD and sCJD agents at 55°C, QuIC-C and RT-QuIC showed shortening of seeding times; however, QuIC-C was again faster than RT-QuIC. These results demonstrated the general applicability of Thermomixers to detect multiple TSE agents in the most extensive comparative study of QuIC assays on thermal mixers to date. The FLUOstar Omega fluorimeter, commonly used for RT-QuIC tests, is a highly versatile device, but it is relatively costly and, in our experience, it is not robust—many hours of vigorous shaking wears on the microplate transport system, requiring periodic refurbishing. Some of these issues can be addressed using a Thermomixer. Other investigators have used Thermomixers at 42°C and reported faster reaction times without reduced sensitivity [16, 20, 21]. Our studies expanded the investigation of Thermomixer QuIC assay to encompass a wider range of TSE infections and furthermore, directly compared PrP^TSE^ amplification across assay platforms and included testing with higher temperatures. We showed that, in addition to 263K scrapie and sCJD seeds, brain homogenates infected with CWD of deer, macaque-adapted vCJD and human vCJD agents were all amplified faster on the Thermomixer, albeit improvements were not the same for all brain seeds.

To explain differences in reaction times with the two test platforms, we verified that temperatures in the wells of the microplates in Thermomixers and FLUOstar Omega were those selected (42°C) and we considered differences in shaking conditions. Both Thermomixers shake plates in single orbits of 3-mm diameter. FLUOstar shakes in a double-orbital movement about 1.35 mm in diameter. This difference is probably important for fibril formation, but we could not rigorously verify effects of this parameter directly. In early studies with 263K hamster brain homogenate, we found that increasing the rate of shaking on the Thermomixer from 700 rpm (our standard conditions) to 1000 rpm did not significantly improve seeding activity, consistent with previously published data [16].

### Hamster versus bank vole recombinant proteins

Truncated Ha-recPrP provided a reproducible and efficient substrate with all TSE brain suspensions tested except for human vCJD brain, which amplified PrP^TSE^ only with truncated BV-recPrP substrate. Truncated BV-recPrP also performed well as substrate with 263K scrapie hamster brain and CWD deer brain suspensions, but results with BV-recPrP were less reproducible than those with Ha-recPrP substrate, especially when samples contained low levels of brain homogenate. Thus, when possible, we used Ha-recPrP as the substrate. Full-length BV-recPrP has been proposed as a universal substrate for RT-QuIC [29]. However, when compared directly in amplification reactions at 42°C, full-length BV-recPrP converted to fibrils more slowly than did truncated BV-recPrP.

### Amplification reactions at 55°C

Increasing the reaction temperature to 55°C, improved assay sensitivity for sCJD agent, as demonstrated by higher SD_50_ values, while reducing assay times. To our knowledge, this is the first report of Thermomixer-C tested at a temperature higher than 42°C [16, 20, 21]. sCJD seeded truncated Ha-recPrP on Thermomixer-C efficiently and reached endpoint in only 8 hours suggesting this assay might be suitable for rapid high-throughput sample screening. We especially focused on detection of CWD agent because of the need for a rapid diagnostic test for cervids. QuIC-C and RT-QuIC had similar sensitivity with QuIC-C reaction although QuIC-C was 12 hours faster. CWD amplification at 55°C did not improve either reaction time or SD_50_ when compared to the results at 42°C (Table 1). It is possible that truncated Ha-recPrP is not the best substrate for CWD tested at higher temperature. Truncated BV-recPrP caused high background fluorescence in negative controls tested at 55°C on both assay platforms. This finding is consistent with those of previous work using full length recombinant human or BV proteins to amplify vCJD PrP^TSE^ at 42°C [16–18, 30, 31]. Our data also indicated that human vCJD PrP^TSE^ efficiently seeded truncated BV-recPrP but only when tested at 42°C.

### Limitations and improvements of QuIC on Thermomixers

Thermomixer-R performed better than Thermomixer-C at 42°C. However, tests on this instrument yielded higher background fluorescence levels in wells containing normal brain tissue compared to tests on Thermomixer-C. On the other hand, reactions on Thermomixer-C required six additional hours to complete and, except with human vCJD and macaque-adapted vCJD brain seeds, QuIC-C yielded 10-fold lower SD_50_ values for the same TSE homogenates, a reduced sensitivity not overcome by increasing the reaction time. QuIC-C performance improved at higher assay temperatures while QuIC-R performance did not.

One limitation of both QuIC assays is that they can be read only at endpoint, while RT-QuIC records the increase in fluorescent signal over time. Kinetic curves generated by the RT-QuIC assay can be informative and especially useful in discriminating false from true positive reactions based on the time when the fluorescent signal first exceeded background. (Early positive reactions are less likely to be nonspecific.) On the other hand, Thermomixers are easier to operate, require no special software, are mechanically sturdier and are cheaper than the FLUOstar Omega. Although a fluorescence plate reader is still needed to read both QuIC-R and C reactions, we conclude that it is preferable to purchase one FLUOstar Omega and several thermomixers than multiple FLUOstar instruments. Thermomixers might offer a promising platform to develop rapid QuIC assays suitable to screen donors of blood and tissues with high throughput, an application for which tracing kinetics of fluorescent signals over time is not necessary.

## References

[1] Will RG. Acquired prion disease: iatrogenic CJD, variant CJD, kuru. Br Med Bull. 2003;66: 255–265. 10.1093/bmb/66.1.255 14522863

[2] Kovacs GG, and Budka H. Prion diseases: from protein to cell pathology Am J Pathol. 2008;172: 555–565. 10.2353/ajpath.2008.070442 18245809 PMC2258253

[3] Büeler H, Aguzzi A, Sailer A, Greiner RA, Autenried P, Aguet M, et al Mice devoid of PrP are resistant to scrapie. Cell. 1993;73: 1339–1347. 10.1016/0092-8674(93)90360-3 8100741

[4] Richt JA, Kasinathan P, Hamir AN, Castilla J, Sathiyaseelan T, Vargas F, et al Production of cattle lacking prion protein. Nat Biotechnol. 2007;25: 132–138. 10.1038/nbt1271 17195841 PMC2813193

[5] Brown P, Brandel JP, Sato T, Nakamura Y, MacKenzie J, Will RG, et al Iatrogenic Creutzfeldt-Jakob disease, final assessment. Emerg Infect Dis. 2012;18: 901–907. 10.3201/eid1806.120116 22607808 PMC3358170

[6] Bonda DJ, Manjila S, Mehndiratta P, Khan F, Miller BR, Onwuzulike K, et al Human prion diseases: surgical lessons learned from iatrogenic prion transmission. Neurosurg Focus. 2016; 41(1):E10 10.3171/2016.5.FOCUS15126 27364252 PMC5082740

[7] Ae R, Hamaguchi T, Nakamura Y, Yamada M, Tsukamoto T, Mizusawa H, et al Update: Dura mater graft-associated Creutzfeldt-Jakob Disease—Japan, 1975–2017. MMWR Morb Mortal Wkly Rep. 2018;67: 274–278. 10.15585/mmwr.mm6709a3 29518068 PMC5844283

[8] Properzi F, and Pocchiari M. Identification of misfolded proteins in body fluids for the diagnosis of prion diseases. Int J Cell Biol. 2013; 839329 10.1155/2013/839329 24027585 PMC3763259

[9] Morales R, Duran-Aniotz C, Diaz-Espinoza R, Camacho MV, Soto C. Protein misfolding cyclic amplification of infectious prions. Nat Protoc. 2012;7: 1397–1409. 10.1038/nprot.2012.067 22743831 PMC4049227

[10] Orrù CD, Groveman BR, Hughson AG, Manca M, Raymond LD, Raymond GJ, et al RT-QuIC assays for prion disease detection and diagnostics. Methods Mol Biol. 2017;1658: 185–203. 10.1007/978-1-4939-7244-9_14 28861791

[11] Atarashi R, Satoh K, Sano K, Fuse T, Yamaguchi N, Ishibashi D. et al Ultrasensitive human prion detection in cerebrospinal fluid by real-time quaking-induced conversion. Nat Med 2011;17: 175–178. 10.1038/nm.2294 21278748

[12] Wilham JM, Orrú CD, Bessen RA, Atarashi R, Sano K, Race B, et al Rapid endpoint quantitation of prion seeding activity with sensitivity comparable to bioassays. PLoS Pathog. 20106; (12):e1001217. 10.1371/journal.ppat.1001217 PMC2996325 21152012

[13] Orrú CD, Bongianni M, Tonoli G, Ferrari S, Hughson AG, Groveman BR, et al A test for Creutzfeldt-Jakob disease using nasal brushings. N Engl J Med. 2014;371: 519–29. 10.1056/NEJMoa1315200 25099576 PMC4186748

[14] Bongianni M, Orrù C, Groveman BR, Sacchetto L, Fiorini M, Tonoli G, et al Diagnosis of human prion disease using Real-Time Quaking-Induced Conversion testing of olfactory mucosa and cerebrospinal fluid samples. JAMA Neurol. 2017;74: 155–162. 10.1001/jamaneurol.2016.4614 27942718

[15] Davenport KA, Hoover CE, Denkers ND, Mathiason CK, Hoover EA. Modified PMCA overcomes RT-QuIC inhibitors in deer saliva to detect CWD prions. J Clin Microbiol. 2018; 56(1): e01243–17. 10.1128/JCM.01243-17 29950332 PMC6113454

[16] Orrú CD, Hughson AG, Groveman BR, Campbell KJ, Anson KJ, Manca M, et al Factors that improve RT-QuIC detection of prion seeding activity. Viruses. 2016; 8(5):140. 10.3390/v8050140 PMC4885095 27223300

[17] Orrú CD, Groveman BR, Hughson AG, Zanusso G, Coulthart MB, Caughey B. Rapid and sensitive RT-QuIC detection of human Creutzfeldt-Jakob disease using cerebrospinal fluid. MBio. 2015 1 20;6(1). 10.1128/mBio.02451-14 PMC4313917 25604790

[18] Franceschini A, Baiardi S, Hughson AG, McKenzie N, Moda F, Rossi M, et al High diagnostic value of second generation CSF RT-QuIC across the wide spectrum of CJD prions. Sci Rep. 2017 9 6;7(1):10655 10.1038/s41598-017-10922-w 28878311 PMC5587608

[19] Favole A, Mazza M, Vallino Costassa E, D'Angelo A, Lombardi G, Marconi P, et al Early and pre-clinical detection of prion seeding activity in cerebrospinal fluid of goats using Real-Time Quaking-Induced Conversion assay. Sci Rep. 2019 4 16;9(1):6173 10.1038/s41598-019-42449-7 30992522 PMC6467873

[20] Atarashi R, Wilham JM, Christensen L, Hughson AG, Moore RA, Johnson LM, et al Simplified ultrasensitive prion detection by recombinant PrP conversion with shaking. Nat Methods. 2008;5: 211–212. 10.1038/nmeth0308-211 18309304

[21] Vendramelli R, Sloan A, Simon SLR, Godal D, Cheng K. ThermoMixer-Aided endpoint Quaking-Induced Conversion (EP-QuIC) permits faster sporadic Creutzfeldt-Jakob Disease (sCJD) identification than Real-Time Quaking-Induced Conversion (RT-QuIC). J Clin Microbiol. 2018; 56(7): e00423–18. 10.1128/JCM.00423-18 29695523 PMC6018336

[22] Cooper JK, Ladhani K, Minor P. Comparison of candidate vCJD in vitro diagnostic assays using identical sample sets. Vox Sang. 2012;102: 100–109. 10.1111/j.1423-0410.2011.01525.x 22126309

[23] Minor P, Newham J, Jones N, Bergeron C, Gregori L, Asher D, et al Standards for the assay of Creutzfeldt-Jakob disease specimens. J Gen Virol. 2004;85: 1777–1784. 10.1099/vir.0.79959-0 15166463

[24] McDowell KL, Nag N, Franco Z, Bu M, Piccardo P, Cervenak J, et al Blood reference materials from macaques infected with variant Creutzfeldt-Jakob disease agent. Transfusion. 2015;55: 405–412. 10.1111/trf.12841 25154296

[25] Bett C, Piccardo P, Cervenak J, Torres JM, Asher DM, Gregori L. Both murine host and inoculum modulate expression of experimental variant Creutzfeldt-Jakob disease. J Gen Virol. 2018;99: 422–433. 29458529 10.1099/jgv.0.001017

[26] Bett C, Grgac K, Lon D, Karfunkle M, Keire DA, Asher DM, et al A heparin purification process removes spiked transmissible spongiform encephalopathy agent. AAPS J. 2017;19: 765–771. 10.1208/s12248-017-0047-y 28116677

[27] Dougherty RM. Animal virus titration techniques In: Harris RJC, editor. Techniques in experimental virology. New York: Academic; 1964 p. 183–186. 10.1016/0042-6822(64)90281-8

[28] McNulty E, Nalls AV, Mellentine S, Hughes E, Pulscher L, Hoover EA, et al Comparison of conventional, amplification and bio-assay detection methods for a chronic wasting disease inoculum pool. PLoS One. 2019 5 9;14(5):e0216621 10.1371/journal.pone.0216621 31071138 PMC6508678

[29] Orrú CD, Groveman BR, Raymond LD, Hughson AG, Nonno R, Zou W, et al Bank vole prion protein as an apparently universal substrate for RT-QuIC-based detection and discrimination of prion strains. PLoS Pathog. 2015; 11(6):e1004983 10.1371/journal.ppat.1004983 26086786 PMC4472236

[30] Levavasseur E, Biacabe AG, Comoy E, Culeux A, Grznarova K, Privat N, et al Detection and partial discrimination of atypical and classical bovine spongiform encephalopathies in cattle and primates using real-time quaking-induced conversion assay. PLoS One. 2017 2 23;12(2):e0172428 10.1371/journal.pone.0172428 28231300 PMC5322914

[31] Peden AH, McGuire LI, Appleford NE, Mallinson G, Wilham JM, Orrú CD, et al Sensitive and specific detection of sporadic Creutzfeldt-Jakob disease brain prion protein using real-time quaking-induced conversion. J Gen Virol. 2012; 93:438–49. 10.1099/vir.0.033365-0 22031526 PMC3352348

